# Supplementary material for: Tourists perceptions of Caribbean islands facing environmental threats before the COVID-19 health crisis: Holbox Island and Archipelago of Bocas del Toro
Source: PLoS One. 2022 Mar 9;17(3):e0262014. doi: 10.1371/journal.pone.0262014 (PMC8906595; doi:10.1371/journal.pone.0262014)
Supplement: Attachment — Submitted filename: Response to Reviewers.docx [file pone.0262014.s001.docx]

Nadia T. Rubio-Cisneros, Ph.D.

Director and Founder

Mar Sustentable Ciencia y Conservación, A.C.

<https://marsustentable.org>

Academic Affiliations

Universidad Autónoma de Nuevo León

Laboratorio de Biología de la Conservación.

Associated Researcher, Center for

Marine Biodiversity and Conservation,

SIO, UCSD

April 8, 2021

PLOS ONE

Editorial Committee

Dear Editorial Committee, it is a pleasure to meet you. I am submitting the reviewers comments for our paper entitled: **¨*The need to address knowledge gaps of Human actions on Caribbean touristic islands facing environmental threats: Tourists' Perceptions of the environment on Holbox Island and archipelago of Bocas del Toro before the COVID-19 health crisis,*** *which is in review for publication* in PLOS ONE.

**Response to Reviewers**

**Reviewer #1:**

The paper is about a very current topic. The research aims and the related methodology are clear and proper. The research is based on a detailed and relevant literature review. The results of the paper have high novelty. I recommend the paper for acceptance in its current form.

**Reviewer #2:**

- The paper focuses on a contemporary and actual topic.
- **The title is too long, should be shortened or compressed somehow.**

*The title was shortened to*

*Tourists perceptions of Caribbean islands facing environmental threats before the COVID-19 health crisis: Holbox Island and Archipelago of Bocas del Toro*

- The abstract is well written, and the keywords are well selected.

**The literature review is too short, should be extended.**

- *The authors added several current references that supported our work ideas. Below I mention them:*

*Abbas, J., Mubeen, R., Iorember, P. T., Raza, S., & Mamirkulova, G. (November 01, 2021). Exploring the impact of COVID-19 on tourism: transformational potential and implications for a sustainable recovery of the travel and leisure industry. Current Research in Behavioral Sciences, 2.*

*Balzan, Mario & Potschin-Young, Marion & Haines-Young, Roy. (2018). Island Ecosystem Services: insights from a literature review on case-study island ecosystem services and future prospects. International Journal of Biodiversity Science, Ecosystem Services & Management. 14. 71-90. 10.1080/21513732.2018.1439103.*

*Brinklow L, Ellsmoor J, Randall J, Rouby M, Sajeva G, Shetye A. Sindico F. COVID 19 Island Insights Series Final Report. University of Strathcyce. Centre for Environmental Law and Governance. 2021; 3-160.*

*Hakim, L. (2020). COVID-19, tourism, and small islands in Indonesia: Protecting fragile communities in the global Coronavirus pandemic. Journal of Marine and Island Cultures, v9n1, 130-141.* [*https://doi.org/10.21463/jmic.2020.09.1.08*](https://doi.org/10.21463/jmic.2020.09.1.08)

[*https://webunwto.s3.eu-west-1.amazonaws.com/s3fs-public/2020-08/SG-Policy-Brief-on-COVID-and-Tourism.pdf*](https://webunwto.s3.eu-west-1.amazonaws.com/s3fs-public/2020-08/SG-Policy-Brief-on-COVID-and-Tourism.pdf)

[*https://www.palcoquintanarroense.com.mx/noticias-de-quintana-roo/habitantes-de-holbox-abandonan-pesca-para-dedicarse-de-lleno-a-actividad-turistica/*](https://www.palcoquintanarroense.com.mx/noticias-de-quintana-roo/habitantes-de-holbox-abandonan-pesca-para-dedicarse-de-lleno-a-actividad-turistica/)

*Jouault, S., Rivera-Nuñez, T., García de Fuentes, A., Xool Koh, M., & Montañez Giustinianovic, A. (2021). Respuestas, resistencias y oportunidades del turismo comunitario en la península de Yucatán frente al COVID-19 y las crisis recurrentes. Investigaciones Geográficas, (104). https://doi.org/10.14350/rig.60240*

*Lopez-Ercilla, Ines, Maria Jose Espinosa-Romero, Francisco J. Fernandez Rivera-Melo, Stuart Fulton, Rebeca Fernández, Jorge Torre, Araceli Acevedo-Rosas, Arturo J. Hernández-Velasco, and Imelda Amador. 2021. "The voice of Mexican small-scale fishers in times of COVID-19: Impacts, responses, and digital divide". Marine Policy. 131: 104606.*

*Mulder, N (coord.), “The impact of the COVID-19 pandemic on the tourism sector in Latin America and the Caribbean, and options for a sustainable and resilient recovery”, International Trade series, No. 157 (LC/TS.2020/147), Santiago, Economic Commission for Latin America and the Caribbean (ECLAC), 2020.*

*Palco Noticias. 2021. Habitantes de Holbox abandonan pesca para dedicarse de lleno a actividad turística. Palcoquintanarroense.com.mx. November 3, 2021.*

*Sheller, M. (2021) Reconstructing tourism in the Caribbean: connecting pandemic recovery, climate resilience and sustainable tourism through mobility justice, Journal of Sustainable Tourism, 29:9, 1436-1449, DOI: 10.1080/09669582.2020.1791141*

*United Nations. 2020. COVID-19 and Transforming Tourism. United Nations.*

- The results are clear, well supported by the methodological toolset.

**Limitations of the study is not described.**

- *The authors modified the previous conclusion* *section to add some examples of limitations that our study can face.*

**Other modifications made to the manuscript are mentioned below.**

- *The figures were updated in the required format using the PASE program.*
- *All the figures developed for this study were from the original idea of first author Nadia Rubio and created with our data sources.*
- *The reference list was updated.*

Sincerely

Nadia Rubio-Cisneros, Ph.D.
